# Semaphorin Signaling in the Development and Function of the Gonadotropin Hormone-Releasing Hormone System

**DOI:** 10.3389/fendo.2013.00133

**Published:** 2013-09-23

**Authors:** Andrea Messina, Paolo Giacobini

**Affiliations:** ^1^INSERM, Laboratory of Development and Plasticity of the Postnatal Brain, Jean-Pierre Aubert Research Center, Unité 837, Lille, France; ^2^School of Medicine, UDSL, Lille, France

**Keywords:** gonadotropin-releasing hormone, reproduction, neuronal plasticity, cell migration, development

## Abstract

The semaphorin proteins are among the best-studied families of guidance cues, contributing to morphogenesis and homeostasis in a wide range of tissue types. The major semaphorin receptors are plexins and neuropilins, however other receptors and co-receptors are capable to mediate signaling by semaphorins. These guidance proteins were originally identified as growth cone “collapsing factors” or as inhibitory signals, crucial for nervous system development. Since those seminal discoveries, the list of functions of semaphorins has rapidly grown. Over the past few years, a growing body of data indicates that semaphorins are involved in the regulation of the immune and vascular systems, in tumor growth/cancer cell metastasis and in neural circuit formation. Recently there has been increasing emphasis on research to determine the potential influence of semaphorins on the development and homeostasis of hormone systems and how circulating reproductive hormones regulate their expression and functions. Here, we focus on the emerging role of semaphorins in the development, differentiation and plasticity of unique neurons that secrete gonadotropin-releasing hormone (GnRH), which are essential for the acquisition and maintenance of reproductive competence in all vertebrates. Genetic evidence is also provided showing that insufficient semaphorin signaling contributes to some forms of reproductive disorders in humans, characterized by the reduction or failure of sexual competence. Finally, we will review some studies with the goal of highlighting how the expression of semaphorins and their receptors might be regulated by gonadal hormones in physiological and pathological conditions.

## Semaphorins and Their Receptors

**Figure 1:**
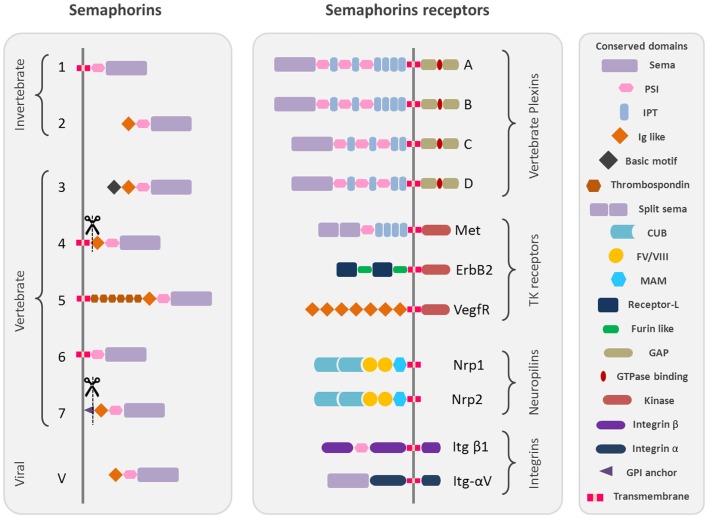
**Schematic representation of the protein structure of semaphorins and their receptors**. Semaphorins are represented in their classification into eighth classes. Class 1 and 2 semaphorins are found in invertebrates. Class 3–7 semaphorins are found in vertebrates. Both semaphorins and plexins are characterized by Sema domains. Additional domains present in semaphorins and plexins include PSI domains (plexin, semaphorin, and integrin) and immunoglobulin (Ig)-like domains. The structural conserved domains are drawn in different shapes and colors as indicated in the figure. Domains abbreviations: PSI, plexin semaphorin integrin; IPT, Ig-like Plexin Transcription factors; Ig-like, immunoglobulin like; CUB, complement C1r/C1s, Uegf, Bmp1; FV/VIII, coagulation factor V/VIII homology like; MAM, meprin like; GPI, glycosylphosphatidylinositol.

**Figure 2:**
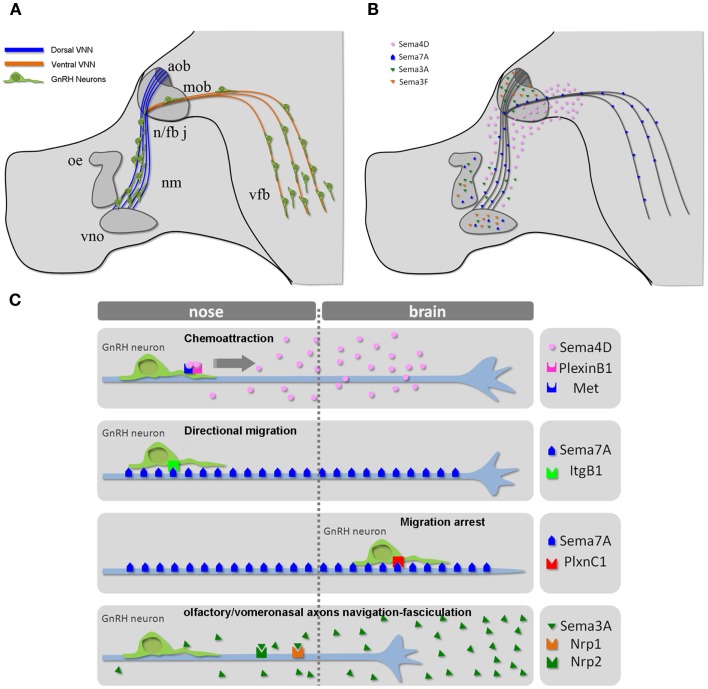
**The GnRH neuronal migratory route**. **(A)** Schematic representation of the head of a mouse embryo at E14.5, depicting the scaffold of vomeronasal/terminal nerve fibers along which GnRH cells migrate from the nose to the ventral forebrain region. **(B)** Schematic depicting the expression pattern of different semaphorins across the GnRH migratory pathway. **(C)** Mechanisms of action of the indicated semaphorins on GnRH cell motility and/or vomeronasal axonal navigation. Abbreviations: VNN, vomeronasal nerve; cx, cerebral cortex; ob, olfactory bulb; nm, nasal mesenchyme; oe, olfactory epithelium; vno, vomeronasal organ; n/fb j, nasal/forebrain junction; aob, accessory olfactory bulb; mob, main olfactory bulb; vfb, ventral forebrain.

Semaphorins are one of the largest protein families of phylogenetically conserved guidance cues and have been extensively studied in a variety of species, including *Caenorhabditis elegans*, *Drosophila*, zebrafish, rodents, and humans. More than 20 semaphorin coding genes have been identified named and grouped into eight classes according to their structural homologies and phylogenetic relationships, including invertebrates (Classes 1 and 2), vertebrates (Classes 3–7), and viral semaphorins (Class V) [Figure 1; (1, 2)].

All semaphorins are characterized by the presence of a *sema* domain, important for dimerization and binding specificity with the receptors, followed by a PSI (plexins, semaphorins, and integrins) domain and a C terminus domain, which confers class specific features (3–5).

The main receptors of semaphorins are the plexins, which are their high affinity receptors. Nine vertebrate plexins have been identified so far, which have been grouped into four subfamilies and show high structural homology with semaphorins as they are characterized by the presence of a *sema* domain (Figure 1) [reviewed by (6)]. Some secreted semaphorins also require the presence of obligate co-receptors, neuropilins (Nrp-1/Nrp-2), which function as the ligand-binding partner in co-receptor complexes for both plexins and vascular endothelial growth factor receptors (VEGFRs) (7, 8) (Figure 1) [reviewed by (9–11)].

Moreover, some semaphorins exert their effect by binding to various holoreceptor complexes associated to plexins. It is the case for different tyrosine kinase receptors like Met (12–14), which also possess a *sema* homologous domain, ErbB2 (15), and the VEGFR2 (16) but also extracellular matrix receptors like alpha and beta integrins (17, 18).

Although the function of semaphorins and their receptors was first acknowledged in the development of the nervous system, these molecules are widely expressed both inside and outside the nervous system [reviewed by (9, 19–22)]. The involvement of semaphorins and plexins in diverse biological processes, such as the development of the nervous and cardiovascular systems, the function of the immune system, and pathological processes such as tumor progression, has also been extensively reviewed (10, 11, 21, 23, 24). Here, we will highlight recent advances in our understanding of the molecular mechanisms underlying the effects of semaphorins on the motility, survival, and axonal plasticity of neurons that secrete gonadotropin-releasing hormone (GnRH).

## Semaphorins in Guidance of GnRH Neuronal Migration

In vertebrates, the GnRH decapeptide regulates the secretion of luteinizing hormone and follicle-stimulating hormone, which govern the onset of puberty, gametogenesis, and estrous cycling, from anterior pituitary gonadotropes (25). During postnatal life, GnRH – secreting neurons are integral members of the hypothalamic-pituitary-gonadal axis. However, during embryonic development, these cells originate from an extracerebral region, namely the nasal placode (26), and migrate to the hypothalamus apposed to olfactory-vomeronasal nerves (VNNs) (27, 28). Alterations in the development of this system or the secretion of GnRH are associated with hypogonadotropic hypogonadism (HH) in humans, a condition characterized by the reduction or failure of sexual competence (29). Unraveling the genetic pathways involved in the regulation of GnRH system development is crucial to understanding the basis of its pathogenesis in human reproductive disorders and formulating novel therapies.

Cell migration plays an essential role in tissue formation during development. The forebrain is one of the most intricate regions of the mammalian brain, and complex migratory movements are required to generate the extraordinary degree of organization observed in this neural structure. Defects in neuronal migration during the development of the forebrain lead to severe learning and cognitive deficits. Understanding how cell migration occurs in the forebrain is therefore essential to discerning the mechanisms underlying its normal and pathological development (30). Abnormal neuronal migration also occurs in diseases that ultimately affect reproduction. For example, in humans, several monogenic disorders leading to idiopathic hypogonadotropic hypogonadism (IHH) are a consequence of the disruption of GnRH neuronal ontogeny/migration (29). The migration of GnRH neurons from the nasal placode, where they are born (27, 28), to the postnatal preoptic area and hypothalamus constitutes one of the best-characterized examples of axonophilic migration in the forebrain (31). To reach their final destination, these neurons migrate along the nasal septum, cross the cribriform plate under the olfactory bulb, and traverse the forebrain, migrating along vomeronasal (VMN) axons (Figure 2A).

The full repertoire of molecular cues regulating the migratory process and precise targeting of GnRH neurons to the hypothalamus has not been completely elucidated. The putative underlying mechanisms involve different classes of signaling molecules, and the list of potential candidates has lengthened during the last decade (26, 32, 33). Another emergent theme from work on various axonal and cell migration guidance cues is that the expression and function of their receptors is under tight molecular control. Such control allows the spatiotemporal regulation of the responsiveness of growth cones to guidance cues. However, even the large number of molecules already identified likely underestimates the complexity of the potential interactions involved. Indeed, GnRH neurons spatially and temporally travel across areas (the nasal region, nasal/forebrain junction, and forebrain; Figure 2) that contain a variety of guidance molecules and factors. In addition, many molecules defy anatomical boundaries by being expressed in multiple areas and may produce different responses depending on the receptor complexes expressed by GnRH neurons as a function of time (embryonic stage) and space (anatomical localization).

During embryonic development, several semaphorins are expressed all along the GnRH migratory route (Figure 2B) (14, 34–41), prompting several investigators to study the potential role of semaphorins in GnRH neuronal migration. Although the regulation of semaphorin receptors remains incompletely understood, recent studies have begun to provide more insight into the sophisticated molecular mechanisms that allow for the diversification and spatiotemporal control of semaphorin responsiveness in developing GnRH neurons and olfactory/vomeronasal axons.

### Semaphorin 4D

Semaphorin 4D (Sema4D) is a membrane-bound semaphorin that can also be proteolytically released (“shed”) into the extracellular space in an active form (42, 43). An intriguing aspect of neural circuit development is that a relatively small number of proteins set up the wiring of a vast number of neuronal connections. Accumulating evidence indicates that mechanisms exist to diversify the effects of guidance proteins, allowing them to mediate this large number of wiring events. How do neurons diversify their responses to semaphorin signaling? One way this goal is achieved is through the ability of individual semaphorins to function as attractive or repulsive guidance cues by activating different receptor complexes on the target cell. One such mechanism has been elucidated for Sema4D, which, in addition to functioning as a collapsing signal for axonal growth cones (44), has previously been shown to induce chemotaxis in epithelial and endothelial cells, and functions as a proangiogenic factor through the coupling its receptor PlexinB1 with the hepatocyte growth factor (HGF) receptor Met tyrosine kinase (12, 13, 45).

PlexinB1 is highly expressed in the developing nasal placode and on olfactory axons and GnRH cells during prenatal development (14), whereas Sema4D is present in the nasal mesenchyme, although its expression is higher in the nasal/forebrain junction and in the developing forebrain. Besides those regions, a recent study showed that olfactory ensheathing cells, which surround GnRH neurons and provide them with an important microenvironment for their migration during development, express Sema4D transcript as well (46).

PlexinB1-deficient mice exhibit a defect in the migration of GnRH neurons that leads to a reduction of GnRH neurons in the adult brain. Postnatal day-3 tissues show a decrease in GnRH neurons in the caudal region of the forebrain and an increase in GnRH neurons in rostral brain regions as a consequence of this migratory defect, which affects the normal embryonic development of these neuroendocrine cells. Interestingly, it has been recently shown that reproduction is also impaired in PlexinB1-ligand, Sema4D knock-out mice as a consequence of a significant decrease in GnRH hypothalamic expression and/or reduced ovarian follicle maturation observed in these mutants (47).

**Figure 3:**
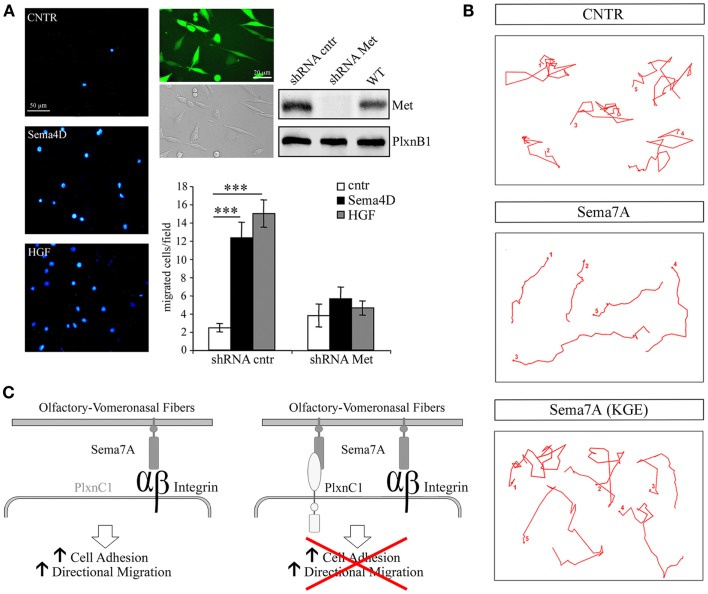
**Semaphorin 4D and 7A directly regulate the motility of GnRH cells**. **(A)** Sema4D promotes directional migration in immortalized GnRH cells, GN11, by coupling Plexin B1 with the activation of the hepatocyte growth factor (HGF) receptor Met tyrosine kinase. Left panel, representative images of a Boyden’s chamber assay showing that GN11 cells migrated through 8 μm membrane pores, attracted by 5 nM Sema4D or 0.5 nM HGF added to the lower chamber. Cells that migrated to the other side of the filter were stained with the nuclear marker DAPI; scale bar, 50 μm. Right panel, GN11 cells were infected with a lentiviral construct encoding for a Met shRNA sequence (shRNA Met) or for a mismatched Met shRNA (shRNA cntr), and for the GFP sequence. Virtually all GN11 cells were infected and expressed GFP. Scale bar, 10 μm. Western blot analysis of Met protein levels in total cell lysates demonstrated that, in Met shRNA – infected cells, Met was silenced. The graph represents the quantitative data of Boyden’s chamber experiments. shRNA cntr cells or shRNA Met cells were treated with 5 nM Sema4D or 0.5 nM HGF. GN11 cells in which Met was silenced were unable to migrate toward a Sema4D gradient. Results are expressed as mean ± SEM. ***P < 0.0001 from two-way ANOVA. **(B)** Representative images from time-lapse videos at different time points show GN11 neurons migrating farther onto Sema7A-coated stripes than onto Sema7A(KGE)-coated stripes as a consequence of increased persistence. The movement of individual cells was recorded during 6 h and plotted. **(C)** Schematic representation of Sema7A function on the GnRH migratory behavior: Sema7A is expressed along the developing olfactory/vomeronasal axons and acts on GnRH neurons increasing adhesion and persistent migration through β1-integrin binding and activation. Expression of PlexinC1 in GnRH neurons switches off their migratory response to Sema7A. Reprinted with permission from (14, 40).

In addition, Sema4D promotes directional migration in immortalized GnRH cells by coupling PlexinB1 with the activation of the Met tyrosine kinase [Figure 3A, reprinted with permission from (14)]. In that study, no abnormalities were observed in the development or organization of olfactory axons, which suggests that the observed migratory defect may be cell-autonomous.

### Semaphorin 7A

The pleiotropic nature of semaphorins is particularly evident for Semaphorin7A (Sema7A), the only glycophosphatidylinositol (GPI)-linked member of the semaphorin family [reviewed in (48)]. Sema7A has been extensively studied in the context of immune function (18) and cancer biology (49–51), and a few reports have addressed its role in neuronal development as well (17, 52–54). Sema7A binds to PlexinC1 to decrease integrin-mediated cell attachment and spreading (49), and its interaction with β1-integrin induces integrin clustering and the activation of MAPK pathways (17). We have also recently reported a role for Sema7A in the regulation of GnRH cell motility (40). Sema7A is expressed along the GnRH migratory route during mouse embryonic development, with high levels of expression observed in the nasal pit, where these cells begin their migratory process, and along the olfactory/vomeronasal axonal scaffold. In addition GnRH cells differentially express the Sema7A receptors β1-integrin and PlexinC1 as a function of their migratory stage: at early stages of their migratory route, GnRH neurons only express β1-integrin, whereas they begin to express PlexinC1 during subsequent developmental stages and at anatomical sites at which these cells terminate their migration (i.e., in the ventral forebrain). Semaphorin signaling is multifaceted, and a subset of these ligands (e.g., Sema4D, Sema6D, and Sema7A) has been shown to elicit integrin activation/cell-substrate adhesion, axonal outgrowth, or cell chemotaxis under distinct conditions (19). Although the molecular mechanisms responsible for these antagonistic activities are not completely understood, they seem to involve distinct signaling pathways that depend on the targeted cells and the different components of semaphorin receptor complexes. For example, Sema7A stimulates the rapid phosphorylation of FAK and ERK1/2 in immortalized GnRH cells and increases the directional migration of these cells in a β1-integrin-dependent manner [Figures 3B,C, reprinted with permission from (40)]. In contrast, the overexpression of PlexinC1 in GnRH neurons leads to reduced migration in the presence of Sema7A (40). This switch may be essential for proper guidance of migrating neurons into the hypothalamus. It is unknown how PlexinC1 expression is induced in migratory GnRH neurons. One possibility is that molecular cues presented by intermediate targets such as the cribriform plate regulate this receptor switch.

*In vivo*, the loss of Sema7A impacts the migration of GnRH neurons, resulting in a significant reduction in the GnRH neuronal population in the brains of adult Sema7A-deficient mice, as well as reduced gonadal size and altered fertility (40). Consistent with these findings, the conditional inactivation of the Sema7A receptor, β1-integrin, in GnRH neurons results in a phenotype that largely resembles that of Sema7A knockdown animals (55). A very interesting aspect of this study is the finding that male reproductive function is not affected by the disruption of β1-integrin signaling in GnRH neurons, in spite of the comparable decrease in the size of the GnRH neuronal population of both male and female *GnRH:Cre;Itgb1^loxP/loxP^* mice when compared with controls (30% reduction). However, the extent of the reduction in GnRH neuronal projections is sexually dimorphic, being milder in male than in female mutant mice (33% reduction in males versus 70% reduction in females). Several reports indicate that gonadal steroids are capable of modulating the expression of different classes of guidance molecules, and thus directing the formation of sexually dimorphic circuits by influencing axonal guidance and synaptogenesis in addition to neurogenesis and cell migration, differentiation, and death (56, 57).

### Semaphorin-3A and -3F

Semaphorins either bind to plexins directly or, in the case of class 3 semaphorins (Sema3s), bind to neuropilins (Nrps), which act as ligand-binding semaphorin co-receptors. The inclusion of additional modulatory co-receptors in the semaphorin receptor complex could provide these receptors with unique signaling properties (21). Two class 3 semaphorins, Sema3A, and Sema3F, are expressed in and around the developing olfactory system (34, 38). The specific co-receptors of these class 3 semaphorins, Nrp-1, and Nrp-2, are expressed on sensory neurons in the main and accessory olfactory epithelia of rodents and zebrafish (34, 36, 38, 58). Members of the PlexinA subfamily are concomitantly expressed in the olfactory system, with the robust expression of PlexinA1 in the vomeronasal organ (VNO) and VNNs (59).

The abnormal accumulation of GnRH neurons has also been observed in the nasal compartment of Nrp-2 knock-out mice, and Nrp-2 has been characterized as the receptor for secreted Sema3F (60). This migratory defect potentially reflects defasciculation problems that affect the olfactory/vomeronasal axons (36, 60) that guide GnRH neurons along their migratory path. Interestingly, consistent with these findings, it has been reported that Nrp-2-deficient mice are typically infertile (36, 61).

Sema3A is a secretory protein with repulsive effects on primary olfactory axons expressing the co-receptor Nrp-1 (37, 62, 63). The role of semaphorins in the navigation of vomeronasal axons and embryonic GnRH cells remains unclear, but previous studies in rodents have shown that migratory GnRH cells are morphologically associated with Nrp-1-immunoreactive axons and are themselves immunoreactive (64, 65). We have recently confirmed these findings in E14.5 mouse embryos and extended them to a 9-week-old human fetus using immunohistofluorescence experiments (66). Notably, the caudal branch of the VNN, which accompanies GnRH cells along their intracerebral path, also expresses Nrp-1.

**Figure 4:**
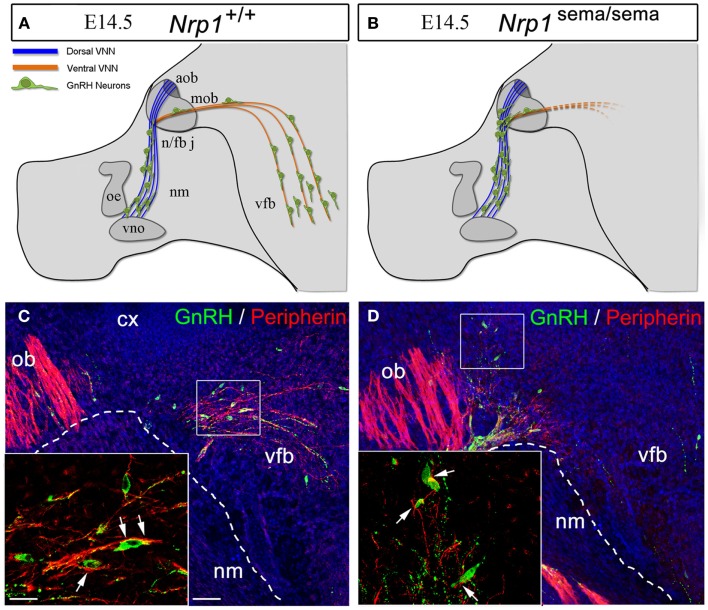
**The defective migration of *Nrp-1^sema/sema^* embryos is not a cell-autonomous trait**. **(A,B)** Defects in olfactory and vomeronasal axons as well as GnRH cell migration in *Nrp-1^sema/sema^* mutant mice. The vomeronasal nerve extends across the medial aspect of the olfactory bulb and projects both dorsally to the accessory olfactory bulb and caudally to the ventral forebrain (vfb). In mutant mice, fibers in the caudal branch are scarce when compared with wild-type mice. **(C,D)** Sagittal sections of the rostral and ventral forebrain regions at E14.5, immunostained for GnRH (green, arrowheads) and the olfactory/vomeronasal marker, peripherin (red). Note the abnormal distribution of GnRH-immunoreactive cells in the *Nrp-1^sema/sema^* mouse [**(D)**, arrowheads]. Other abbreviations: VNN, vomeronasal nerve; cx, cerebral cortex; ob, olfactory bulb; nm, nasal mesenchyme; oe, olfactory epithelium; vno, vomeronasal organ; n/fb j, nasal/forebrain junction; aob, accessory olfactory bulb; mob, main olfactory bulb; vfb, ventral forebrain. Scale bars: **(C,D)**, 50 μm; insets, 20 μm. Adapted from Ref. (66), with permission.

In a series of genetic mouse models and *in vitro* experiments, it has recently been shown that the development of the GnRH system relies on Sema3A signaling through Nrp-1 and Nrp-2 (64–66). Mice lacking Sema3A or semaphorin signaling through both Nrps show phenotypical features associated with fetal X-linked Kallmann’s Syndrome (KS) (67), i.e., the accumulation of GnRH neurons and vomeronasal axons at the dorsal surface of the cribriform plate (64–66). Additionally, DiI axonal labeling at E14.5 shows the abnormal projection of the VNN into the ventral forebrain in mutant embryos lacking a functional semaphorin-binding domain in Nrp-1 [Nrp-1^sema/sema^; Figures 4A–D, Figures 4C,D are reprinted with permission from (66)]. Considerable abnormal cell migration is seen in these mutants as a consequence of the aberrant projections of the VNN (66). Incidentally, in conditional mutant mice lacking Nrp-1 in only GnRH neurons (*GnRH:cre; Nrp-1^loxP/loxP^* mice), the distribution of these cells between the nose and the brain at E14.5 is normal, as is the number of these cells in the adult brain (66), thus confirming that the defective migration seen in Nrp-1^sema/sema^ embryos is not a cell-autonomous trait but rather a consequence of the misrouting of the VNNs into the ventral forebrain (Figures 4A–D).

In addition to binding secreted semaphorins, the Nrps are recognized co-receptors for vascular endothelial growth factor (VEGF) (68–70), which is a key regulator of angiogenesis in health and disease (71, 72) and a critical molecule for vascular development (73, 74). It has recently been shown that Sema3A-mediated axonal guidance takes place in cooperation with the alternative Nrp-1 ligand, VEGF164, which mediates neuronal survival through neuronal, but not endothelial, Nrp-1 expression, to ensure that migrating GnRH neurons reach the brain (64). This study challenges two widely held models, which suggest that (1) the lack of a catalytic intracellular domain forces Nrp-1 to use KDR, the main VEGF receptor in blood vessels, as a co-receptor for the transduction of VEGF signals (75) and (2) KDR is the main VEGF receptor involved in the promotion of neuronal survival (76–78). In contrast to these models, Cariboni and colleagues have observed that VEGF164 signaling in GnRH neurons does not require KDR, which instead promotes the survival of migrating GnRH neurons via the co-activation of ERK and AKT signaling pathways through Nrp-1.

It is widely accepted that the vascular role of Nrp-1 reflects the loss of VEGF rather than Sema3A signaling, as supported by the fact that mice expressing a mutant Nrp-1 that is only capable of binding VEGF, but not semaphorins, do not exhibit vascular defects (79). Whether neuronal VEGF acts through unconventional and previously unsuspected signaling pathways is still a matter of investigation.

## Semaphorin Mutations in Human Forms of Reproductive Insufficiency

The use of animal models has been tremendously helpful in showing that semaphorin signaling is crucial for the development, migration, survival, and maturation of the GnRH system, and that various forms of reproductive insufficiency involve mutations in several genes of the semaphorin family.

Isolated GnRH deficiency, termed central hypogonadism, is an inheritable yet underestimated disorder characterized by absent or incomplete sexual maturation and low circulating gonadotropin and sex steroid levels but otherwise normal pituitary function/imaging (80, 81). The disease can be associated with either a normal sense of smell (normosmic idiopathic central hypogonadism, nICH) or with anosmia/hyposmia (KS), and in both cases, anomalies in the embryonic development of the olfactory and GnRH neuronal system have been described.

Mutations affecting at least 17 disease-genes have been associated with the onset of nICH/KS (82): anosmin-1 (or KAL1), fibroblast growth factor receptor-1 (FGFR1), fibroblast growth factor 8 (FGF8), GnRH-1 and GnRH receptor (GnRHR), nasal embryonic LHRH Factor (NELF), G-protein-coupled receptor 54 (GPR54)/kisspeptin receptor (KISS-R) and kisspeptin1 (KISS-1), prokineticin-2 (PROK2) and prokineticin receptor-2 (PROKR2), chromodomain helicase DNA-binding protein-7 (CHD7), neurokinin-B (TAC3) and neurokinin-B receptor (TAC3R), heparan sulfate 6-*O*-sulfotransferase 1 (HS6ST1), WD protein interacting with EMX1 transcription factor (WDR11), SEMA3A and more recently SOX10 (83). However, these mutations account for only 30% of nICH/KS patients (84), and current efforts thus concentrate on the identification of other genes that could contribute to these disorders.

One research strategy is based on the histopathological examination of targeted mutant mice that could reproduce the human KS phenotype. Using this strategy, we have recently shown that *Nrp-1^sema/sema^* mutant mice, which lack the semaphorin-binding domain in Nrp-1, exhibit a KS-like phenotype, and genetic evidence shows that insufficient semaphorin-3A signaling contributes to the KS phenotype in humans (66). We identified eight different mutations in the Sema3A gene in 24 of the 386 KS patients studied (i.e., approximately 6%). Interestingly, the mutations were consistently observed in the heterozygous state, and five patients carried additional heterozygous mutations in PROKR2, PROK2, KAL1, or FGFR1. These results thus identify Sema3A as a novel contributory gene in KS and further substantiate the oligogenic pattern of inheritance in this developmental disorder (85, 86).

Young and colleagues have also reported a large deletion in Sema3A associated with KS in the heterozygous state in two siblings and their clinically affected father (87). However, these findings do not support autosomal dominant Mendelian inheritance in the analyzed family, and suggest that another, as yet unidentified, genetic hit is associated with the Sema3A haploinsufficiency that results in this disease phenotype (66).

A mutation in another class 3 semaphorin, Sema3E, has also been reported in an individual affected by CHARGE syndrome (88). CHARGE syndrome, which includes eye coloboma, heart malformations, atresia of the choanae, retardation of growth/development, genital anomalies, and ear abnormalities, is caused by mutations in CHD7 (89). CHARGE patients may display anosmia and/or hypogonadism, which are features that overlap with IHH and KS. Similarly, some IHH/KS patients also display partial CHARGE features. Therefore, it has been hypothesized that IHH/KS represents a milder allelic variant of CHARGE syndrome, which has been supported by the identification of heterozygous CHD7 mutations in nICH/KS individuals (89, 90).

## Semaphorins and the Plasticity of Adult Hormone Systems

**Figure 5:**
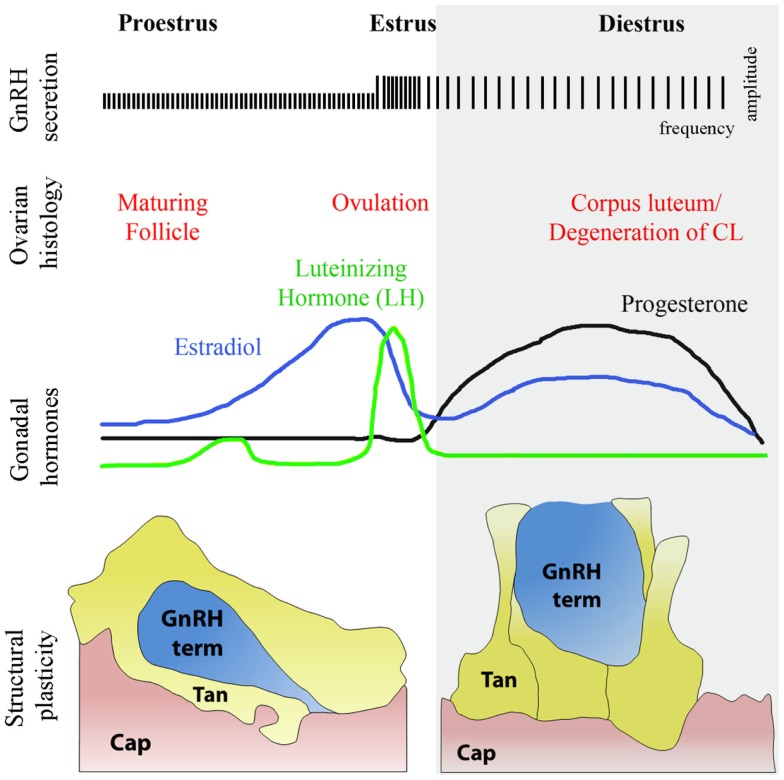
**Structural plasticity of the GnRH nerve terminals and tanycytic end-feet in the median eminence of the hypothalamus during diestrus and proestrus**. The schematic highlights the causal relationships during the different phases of the ovulatory cycle with changes respectively in GnRH secretion, ovarian histology, and circulating sex hormones levels. Changes in circulating gonadal steroids are responsible for the neuro/glial structural plasticity of the median eminence. During proestrus, GnRH nerve endings (blue) sprout toward the basal lamina delineating the pericapillary space (pink, Cap), with which they eventually make direct contact, while tanycytes retract. In diestrus, under conditions of low gonadotropin output, GnRH-secreting axon terminals are distant from the pericapillary space and tanycytes enwrap GnRH nerve endings, thus impairing access of the neurohormone to the pituitary portal circulation.

Blood vessels and axons employ similar mechanisms and follow common guidance cues for growth and navigation during embryonic development (91, 92). Blood vessels influence axonal trajectories to permit them to reach the appropriate destinations (93). In the adult brain, blood vessels communicate with neurons and glia to meet physiological demands (94). Endothelial cells are ideally positioned to sense peripheral inputs and to convey signals that could influence neuronal structure and synaptic plasticity. However, whether these cells are capable of influencing axonal plasticity in the mature central nervous system remains largely unknown. Recent evidence suggests that semaphorins are constitutively expressed in the postnatal brain and may be involved in neuronal plasticity and nervous system physiology (95). Semaphorins are also expressed in endothelial cells during vascular development (96). Interestingly, as elaborated in the previous sections, semaphorins act as guidance factors during the migration of GnRH neurons (14, 40, 60, 65, 66), which retain a high degree of plasticity in the mature brain (97). As the final common element in the central control of gonadotropin secretion, GnRH neurons, which project into the hypothalamic median eminence and release the neurohormone into a specialized capillary network for delivery to the anterior pituitary (98), are affected by numerous regulatory homeostatic and external factors to provide levels of fertility appropriate to the organism. For instance, GnRH neurons undergo extensive axonal growth toward the vascular wall during critical time windows in adulthood, such as at the onset of the preovulatory surge, when massive GnRH release occurs to induce ovulation (97) (Figure 5). They are thus an ideal system for analyzing the complex relationships involved in neurosecretion and morphological plasticity as well as the function of specific molecules in the homeostasis of the adult nervous system (97, 99, 100). Emerging data from our group shows that this periodic sprouting of GnRH axon terminals is under the control of specific semaphorins, which thus play a pivotal role in orchestrating the central control of reproduction (101, 102). Altogether, these findings raise the intriguing possibility that semaphorins may play important and unexpected roles in the adult neural plasticity that underlies key physiological processes, such as reproduction.

## Regulation of Semaphorins Expression in the Pituitary-Gonadal Axis

The expression of semaphorins, plexins, and neuropilins is consistent with a prominent role for these molecules in the development and function of the hypothalamic-pituitary-gonadal system. The pituitary consists of three lobes: the neural lobe, the intermediate lobe, and the anterior lobe. The neural lobe is derived from diencephalic tissue, and the anterior and intermediate lobes from an invagination of the oral ectoderm called Rathke’s pouch. During pituitary development, the expression of Sema7A is most pronounced in Rathke’s pouch, whereas it is strongly expressed in the intermediate and anterior lobes at later developmental stages (39). In contrast, PlexinC1 is largely confined to the neural area of the pituitary, with only weak expression observed in the intermediate lobe at the late embryonic stage and not at all at the postnatal or adult stages.

Outside the central nervous system, fluctuations in reproductive hormones play a well-established role in the maintenance of body homeostasis. For instance, *ex vivo* data have shown that Sema4D is involved in the maturation of ovarian follicles in mice (103). Sema4D and its receptor PlexinB1 are both expressed in the mouse ovary *in vivo* mostly in the granulosa cells. The finding that Sema4D increases after hormonal treatment suggests follicular auto-regulation of the plexin/semaphorin system. However, the mechanism by which Sema4D/PlexinB1 system affects follicular growth and steroidogenesis requires further clarification.

An analysis of Sema4D^−/−^ and PlexinB1^−/−^ mice and of mice expressing a dominant-negative form of RhoA specifically in osteoblasts has revealed an osteosclerotic phenotype in these mutants (104). Interestingly, this phenotype is strictly dependent on ovarian function (circulating estrogens) as an ovariectomy suppresses the bone resorption phenotype in Sema4D^−/−^ mice (47), thus suggesting that Sema4D expression might be under the control of circulating gonadal hormones. This hypothesis is supported by previous studies, which have demonstrated that semaphorins and their receptors expressions are regulated by steroid hormones in physiological and pathological conditions. Pavelock and coworkers provided direct evidence for the expressions of Nrp-1 and Nrp-2 transcripts in uterine tissue and they have shown that the levels of these neuropilins mRNAs are respectively progesterone- and estrogen-dependent (105). The regulated expression and differential localization of Nrp-1 and Nrp-2 in the rat uterus suggest that these receptors may participate in hormonally regulated changes occurring throughout the female reproductive cycle (105).

Class 3 semaphorins, Sema3B and Sema3F, are secreted proteins that regulate angiogenesis, tumor growth, and metastasis by binding to their transmembrane receptor complex consisting of plexins and neuropilins (23). Interestingly, a significantly reduced expression of Sema3B (83 kDa), Sema3F (90 kDa), and plexin-A3 was observed in human ovarian cancer cell lines when compared with normal human ovarian surface epithelial cells (106). The treatment of tumor cells with luteinizing hormone, follicle-stimulating hormone, estrogen, and progesterone induced significant changes in Sema3B and Sema3F expression (106), implying that gonadotropin- and/or estrogen-mediated maintenance of Sema3 expression could control ovarian cancer angiogenesis and metastasis. These findings have important clinical ramifications as they may explain why some of these tumors become more prevalent around the perimenopausal and postmenopausal periods of women. The same research group has also provided compelling evidence that Sema3B, Sema3F, and plexin-A3 were expressed strongly in normal endometrial tissues, whereas grade-dependent decreases were found in endometrial carcinomas (107). Moreover, treatment of cancer cells with progesterone and 1,25-dihydroxyvitamin D3 [1,25(OH)2D3] for a period of 72 h induced a significant upregulation of these semaphorins as well as inhibited growth of cancer cells by increasing caspase-3 activity (107).

Besides hormonal signaling, recent studies have demonstrated that different classes of growth factors might also regulate semaphorins’ expression. Fibroblast growth factor 8 (FGF8) has been shown to repel midbrain dopaminergic neuron (mDAN) axons that extend through the diencephalon (108). This repulsion is mediated by Sema3F, since the expression of Sema3F is up-regulated by ectopic expression of FGF8 and Sema3F repels mDAN axonal growth (108).

Another study showed that Sema7A expression is stimulated by transforming growth factor-beta1 (TGF-β1) in the murine lung, with Sema7A being a critical regulator of tissue remodeling in TGF-β1-induced pulmonary fibrosis (109).

It is likely that the some growth factors might also be under the control of hormonal signaling. Whether these hormones regulate semaphorins expression directly and/or indirectly through growth factors modulation in neuronal and non-neuronal adult tissues will require future investigation.

## Conclusion

Semaphorin signaling plays a pivotal role in nervous system development and neural network assembly, and has been shown to influence cellular morphology in a large variety of systems. In this review, we have focused on factors and receptors belonging to this diverse family of guidance cues and their influence on the development and function of multifaceted hormonal tissues, with an emphasis on neuroendocrine systems. These activities underlie complex developmental processes, such as the migration of neurons that control fertility from the nose to the brain, the wiring of neuroendocrine networks and the genetic bases of some forms of reproductive disorders. The recent identification of mutations in semaphorin genes in patients with developmental neuroendocrine deficiencies associated with infertility illustrates the importance of semaphorins in this process. Furthermore, semaphorin expression persists in adulthood, and it has been proposed that these signals play an important role in the plasticity of the neuroendocrine systems that defend homeostatic set points to enable the survival of individuals and species. The identification of semaphorins and their associated receptors as participants in both the development and functional plasticity of hormone systems creates new avenues of investigation in endocrinology and neuroendocrinology.

## Conflict of Interest Statement

The authors declare that the research was conducted in the absence of any commercial or financial relationships that could be construed as a potential conflict of interest.
